# Genitourinary cancers updates: highlights from ASCO 2023

**DOI:** 10.1186/s13045-023-01511-8

**Published:** 2023-11-21

**Authors:** Qian Qin, Hollie Sheffield, Sean M. Taasan, Andrew Z. Wang, Tian Zhang

**Affiliations:** 1 Division of Hematology and Oncology, Department of Internal Medicine, UT Southwestern Medical Center, Dallas, TX 75390 USA; 2 Department of Radiation Oncology, UT Southwestern Medical Center, Dallas, 75235 USA

## Abstract

Significant scientific advances in immunotherapy and targeted therapy approaches have improved clinical outcomes and increased treatment options for patients with genitourinary (GU) malignancies. We highlight the clinical trial developments released at the ASCO 2023 annual meeting, including PARP inhibitors for prostate cancer, antibody drug conjugates and fibroblast growth factor receptor inhibitors for urothelial cancer, and HIF2a inhibitors for renal cell carcinoma. Novel agents such as bispecific antibodies, chimeric antigen receptor T-cells, and radiopharmaceuticals are currently in early phase development and also have high potential impact for the GU cancer landscape. With more treatment options, the field will need to define best treatment sequencing to optimize outcomes for each patient.

## Introduction

Therapeutic advances in genitourinary (GU) malignancies have progressed rapidly. Prostate cancer has seen multiple combination treatments in upfront metastatic hormone sensitive disease, as well as the addition of PARP inhibitors for castration resistant prostate cancer with homologous recombination repair defects. In urothelial cancer, the advent of antibody drug conjugates and fibroblast growth factor receptor inhibitors have added multiple therapeutic options in the advanced setting, and many are under investigation in the localized setting. In kidney cancer, combination regimens of immune checkpoint inhibitor (IO) with or without vascular endothelial growth factor receptor-tyrosine kinase inhibitors (VEGFR-TKIs) have become standard of care, and novel agents such as HIF2a inhibitors hold much promise. We summarize recent therapeutic updates in GU cancers at the ASCO 2023 annual meeting.

## Prostate

Several trials highlighting novel treatments under investigation (including PSMA-directed radionuclide therapy, PARP inhibitors, immunotherapy, bispecific antibodies, and CAR T-cells) were featured (Table 1). In addition, further data from two previously published major phase 3 trials (PEACE-1, TALAPRO-2) with potentially practice-changing implications were presented [1, 2].

**Table 1:** Novel regimens under investigation—prostate cancer highlights from ASCO 2023

Reference number	Phase	Intervention	*n*	Population	Notes
[3] (PSMAddition)	3 (active)	^177^Lu-PSMA-617 every 6 weeks up to 6 cycles + SOC (ARPI + ADT)	1126 (planned)	mCSPC with PSMA positive disease and without rapid tumor progression, first-line	Primary endpoint: rPFS PSMA positivity determined by ^68^Ga-PSMA-11 PET/CT
[4] (BXCL701)	2b (active)	BXCL701 (d1-14) + pembrolizumab (d1) given over 21 day cycles	60 (planned)	mCRPC with small cell neuroendocrine phenotype, progression on at least one line of prior cytotoxic therapy	Primary endpoint: response rate BXCL701 is a dipeptidyl peptidase inhibitor theorized to trigger inflammasome and affect immune priming Comparison arm is BXCL701 monotherapy
[5] (PRIME-CUT)	2	Docetaxel (two cycles) followed by cemiplimab (d1) given over 21 day cycles	20	mCSPC, first-line therapy	Primary endpoint: undetectable PSA at 6 months Primary endpoint not met (10% compared to prespecified historical rate of 32%)
[6] (MAVERICK)	2 (active)	Abivertinib (200 mg BID) + abiraterone (1000 mg qd)	100 (planned)	mCRPC with HSD3B1(1245C) allele, two cohorts (abiraterone-naive and abiraterone-progressing)	Primary endpoint: 6-month rPFS HSD3B1(1245C) allele present in up to 50% of patients. Missense in corresponding enzyme causes up-regulation in rate-limiting step of extragonadal androgen biosynthesis Abivertinib is a TKI that inhibits phosphorylation of the above enzyme
[7] (talazoparib/temozolomide)	1b/2 (phase 1b accrual complete, phase 2 active)	Talazoparib (d1-6) + temozolomide (d2-8) given over 28 day cycles	13 (phase 1) 55 (phase 2, planned)	mCRPC without DNA damage repair mutations, progression on at least one ARPI	Primary objective (phase 1): safety. Hematologic toxicity was identified as the DLT Primary endpoint (phase 2): overall response rate
[8] (COMRADE)	1/2 (phase 1 accrual complete,, phase 2 active)	Olaparib (200 mg BID) + radium-223 (every 4 weeks × 6 cycles)	12 (phase 1) 133 (phase 2, planned)	mCRPC with ≥ 2 bone metastasis, any line	Primary objective (phase 1): determination of MTD Primary endpoint (phase 2): rPFS Any HRR status allowed in this study
[9] (DUET)	1/2 (active)	Radium-223 (weeks 0, 4, 8) alternating with ^177^Lu-PSMA-617 (weeks 6, 12)	8 (planned)	mCSPC with low volume (2–5 bone metastases, 0–3 lymph node metastases) and PSA doubling time < 6 months, occurring following prior curative intent treatment (surgery or radiation)	Primary objective: feasibility/safety of radium-223 alternating with ^177^Lu-PSMA-617
[10] (tildrakizumab)	1/2 (active)	Tildrakizumab + abiraterone acetate	10 (phase 1, planned) 25 (phase 2, planned)	mCPRC, prior progression on ADT + abiraterone and/or enzalutamide	Primary objective (phase 1): MTD and RP2D determination Primary endpoint (phase 2): overall response rate Tildrakizumab is an IL-23 inhibitor. IL-23 blockade has been shown to reverse resistance to ARPI in vivo
[11] (CABIOS)	1b (active, interim analysis)	Cabozantinib [20 mg or 40 mg qd] + nivolumab (every 4 weeks) + abiraterone (1000 mg qd)	17 (interim)	mCSPC, de novo or recurrent without prior abiraterone or docetaxel	Primary endpoint: safety and tolerability At median follow-up of 12.8 months during interim analysis, 9 patients remain on study with 1 withdrawal, 4 discontinuations due to toxicity, and 3 discontinuations due to progression
[12] (LuPARP)	1 (active, interim analysis)	^177^Lu-PSMA-617 (every 6 weeks × 6 cycles) + olaparib	48 (planned)	mCRPC and high PSMA expression (SUVmax ≥ 15 at site of disease and ≥ 10 at other sites), prior therapy allowed	Primary objective: establishing DLT and RP2D Interim analysis showing most common G1-2 toxicity was xerostomia (83%), nausea, (62%), fatigue (34%) and constipation (31%). 18/29 patients in interim analysis with at least 50% reduction in PSA
[13] (ProSperA)	1 (active)	CC-1	24–42 (dose-escalation, planned) 14 (dose expansion, planned)	Low-risk biochemical recurrence after prostatectomy or radiation	Primary objective: identification of MTD and RP2D CC-1 is a PSMAxCD3 bispecific antibody. CC-1 is also being evaluated in a separate phase I trial for mCRPC
[14] (PSCA CAR-T)	1	PSCA-targeted 4-1BB co-stimulated CAR-T	14	mCRPC, progression after at least 1 prior ARPI	Primary objective: establishing DLT (cystitis) Lymphodepleting chemotherapy was necessary for greater CAR T expansion; lower doses of lymphodepleting chemotherapy reduced toxicity without clear impact on expansion

*PSMA* Prostate-specific membrane antigen, *SOC* Standard of care, *ARPI* Androgen receptor pathway inhibitor, *ADT* Androgen deprivation therapy, *mCSPC* Metastatic castration sensitive prostate cancer, *rPFS* Radiographic progression free survival, *mCRPC* Metastatic castration resistant prostate cancer, *TKI* Tyrosine kinase inhibitor, *HRR* Homologous recombination repair, *MTD* Maximum tolerated dose, *DLT* Dose-limiting toxicity, *RP2D* Recommended phase 2 dose, *PSCA* Prostate stem cell antigen, *CAR-T* Chimeric antigen receptor T-cell

Late-breaking data from PEACE-1 evaluating the impact of radiotherapy in first-line patients with de novo metastatic castration sensitive prostate cancer (mCSPC) showed a statistically significant increase in the co-primary endpoint of rPFS in patients with low-volume mCSPC who received standard of care (SOC) + abiraterone + radiotherapy compared to SOC + abiraterone (median 7.5 versus 4.4 years, *p *= 0.02) [15]. There was no statistically significant rPFS benefit when radiotherapy was added to SOC alone. The co-primary endpoint of overall survival (OS) was not statistically significantly improved between the cohorts, although it is difficult to show an OS improvement in this patient population. Secondary endpoints of castration resistance-free survival and time to serious genitourinary events were improved with the addition of radiotherapy to SOC (± abiraterone) in both the low-volume and overall cohorts. Rates of toxicity appeared similar between groups receiving radiotherapy versus those that did not. Overall, these data suggest a role for radiotherapy in combination with systemic treatment (particularly in regimens containing abiraterone) in patients with low-volume de novo mCSPC.

New data from TALAPRO-2 were presented, evaluating enzalutamide + talazoparib versus enzalutamide + placebo as first-line therapy in patients with metastatic castration resistant prostate cancer (mCRPC) in a cohort of patients specifically selected for homologous recombination repair (HRR) gene alterations, including 169 patients from the original TALAPRO-2 cohort and 230 patients enrolled later [16]. The most common HRR gene alterations present were *BRCA2* (~ 30–35% of patients) followed by *ATM*, *CDK12*, *CHEK2* (~ 15–25% each) and *BRCA1* (~ 5%). At a median follow-up of ~ 17 months, talazoparib significantly improved the primary endpoint of rPFS compared to placebo (median NR vs. 13.8 months, HR 0.45; 95% CI 0.33–0.81; *p *< 0.0001). This benefit was greater in patients with a *BRCA1/2* alteration (HR 0.20; 95% CI 0.11–0.36; *p *< 0.0001) as compared to those without (HR 0.72; 95% CI 0.49–1.07; *p *= 0.10). Although OS trended toward improvement, the data was not yet mature at time of analysis. The primary toxicity noted with the addition of talazoparib was increased cytopenias, particularly grade 3/4 anemia which occurred in ~ 40% of patients on talazoparib and only 4.5% in the placebo group. Dose interruptions (67% versus 37%) and dose reductions (56% vs. 6%) were more common in the talazoparib group compared to placebo, however rates of discontinuation due to adverse events were similar (10% versus 7%). Overall, this update from TALAPRO-2 provides the basis for the FDA approval of first-line therapy for mCRPC with HRR alterations of enzalutamide with talazoparib. This joins the two other FDA approvals in 2023 of niraparib-abiraterone (based on MAGNITUDE) and olaparib-abiraterone (based on PROPEL), both for BRCA1/BRCA2 mutated mCRPC.

## Urothelial carcinoma

Antibody drug conjugates (ADC) and fibroblast growth factor receptor (FGFR) inhibitors have improved outcomes in urothelial cancer, and trials combining immunotherapy with both drug classes were presented at ASCO 2023 (Table 2).

**Table 2:** Novel regimens under investigation—urothelial cancer highlights from ASCO 2023

Reference number	Phase	Intervention	*n*	Population	ORR	mPFS	mOS	Safety
[17] EV103	1b/2	Enfortumab vedotin + Pembrolizumab	45	Patients with metastatic urothelial carcinoma who were cisplatin ineligible	73.3% (58.1–85.4)	12.7 mos	26.1 mos	64.4% with Grade III toxicity, primarily elevated lipase, maculo-papular rash, and fatigue
[18] THOR	3	Erdafitinib versus chemotherapy (investigator’s choice) in patients with FGFRalt	266	Patients with urothelial carcinoma with FGFRalt on 2nd or 3rd line therapy	45.6 versus 11.5	5.6 versus 2.7	12.1 versus 7.8 mos	Serious treatment-related events 13% with Erdafitinib versus 24% in chemotherapy arm
[19] Norse	2	Erdafitinib compared to Erdafitinib + Cetrelimab in metastatic urothelial carcinoma with presence of FGFRalt	87	Patients with metastatic urothelial carcinoma with FGFRalt and are cisplatin ineligible	54.5% in combination versus 44.2% in ERDA alone	10.97 mos versus 5.62 mos	-	1 death in combination group from pulmonary failure, otherwise no added toxicity with addition of Cetrelimab to ERDA

Enfortumab vedotin (EV) was the first ADC that received FDA approval in 2019 for patients with locally advanced or metastatic urothelial carcinoma (mUC) who had previously received IOs and platinum-containing chemotherapy, based on the EV-301 trial [20]. Since its approval, EV has had clear implications in the treatment guidelines as a second or third line option for patients with mUC. EV-103 is a phase 1b/2, open-label, multiple cohort study for mUC, and patients enrolled in cohort A had cisplatin-ineligible mUC treated with EV and pembrolizumab in the first-line setting [17]. The objective response rate was 73.3% (95% CI 58.1–85.4), and the disease control rates were 84.4% (95% CI 70.5–93.5). With prolonged follow-up, progression-free survival of this cohort was 41.4% at 2 years, with median OS at 26.1 months. The treatment-related adverse events were primarily elevated lipase (17.8%), maculopapular rash (11.1%), and fatigue (11.1%). These data set up the EV-302 trial, a randomized phase 3 trial of EV-pembrolizumab compared with standard platinum-based chemotherapy in first-line mUC. EV-pembrolizumab improved OS (mOS 31.5mo vs. 16.1mo, HR 0.47, 95% CI 0.38–0.58, *p *< 0.00001) as well as PFS (mPFS 12.5mo vs. 6.3mo, HR 0.45, 95% CI 0.38–0.54, *p *< 0.00001) [21].

Separately, data from TROPHY-U-01 (phase II, open-label, multiple cohort study) led to the approval of sacituzumab govitecan (ADC against trop-2-expressing tumor cells) in patients with mUC refractory to first line platinum-based chemotherapy and IOs [22]. Finally, disitamab vedotin is the newest ADC targeting HER2 that is being studied in phase-II clinical trials RC48, which have positive preliminary results.

A significant percentage of about 10–20% of urothelial carcinomas harbor FGFR alterations. Erdafitinib is a FGFR inhibitor that was studied first in a phase 2 trial and then validated in THOR, a phase 3 randomized controlled trial comparing erdafitinib to the standard of care chemotherapy (docetaxel vs. vinflunine) [18]. Erdafitinib improved OS (median 12.1 vs. 7.8 months), PFS (median 5.6 months vs. 2.7 months), and ORR (45.6% vs. 11.5%) when compared to chemotherapy. Erdafitinib also had fewer serious treatment-related adverse events, 13.3% versus 24.1%, and fewer deaths, 1 versus 6, when compared to the chemotherapy control group. Primary grade 3 or 4 toxicities with erdafitinib were hyperphosphatemia, diarrhea, stomatitis, and palmar-plantar erythrodysesthesia.

Additionally, the Norse phase 2 study by Siefker-Radtke et al. explored the addition of cetrelimab (PD-1 inhibitor) to erdafitinib in the first line setting in patients who are both FGFR mutated and cisplatin ineligible [19]. There was increased ORR of 54.5% at 12 months in the combination group compared to 44.2% in the erdafitinib alone group. The median PFS was 10.97 months in the combination group compared to 5.62 months in the erdafitinib alone arm.

## Renal cell carcinoma

In the treatment of clear cell renal carcinoma (ccRCC), there are five first-line doublet options in the treatment naïve and multiple single agent/double options in the refractory setting available. However, the bases for these approved therapies are IO, VEGFR-TKI, and/or mammalian target of rapamycin (mTOR) inhibitor. Additional agents and therapeutic combinations are urgently needed, and ASCO 2023 highlighted several currently under investigation.

The optimal therapeutic option for patients who have progressed on IO therapy has been debated, and the phase III CONTACT-03 trial evaluated cabozantinib 60 mg PO daily (*n *= 259) with or without atezolizumab 1200 mg IV every 3 weeks (i.e., IO rechallenge, *n *= 263) in patients with advanced RCC and radiographic progression on or after IO therapy [23, 24]. At a median follow-up of 15.2 months, when comparing atezolizumab-cabozantinib to cabozantinib, no statistically significant differences were observed in the primary endpoints of PFS (median 10.6 vs. 10.8 months, HR 1.02, 95% CI 0.83–1.28, *p *= 0.78) or OS (median 25.7 months vs. not evaluable, HR 0.94, 95% CI 0.70–1.27, *p *= 0.69). Increased toxicity was observed in the combination arm, which discourages the sequential use of cabozantinib-atezolizumab immediately after IO progression.

In terms of novel agents, preliminary results from arm B5 of the phase I/II KEYMAKER-U03B study were reported. In the U03B trial, belzutifan 120 mg PO daily (HIF-2a inhibitor) plus lenvatinib 20 mg PO daily was given to 30 patients with advanced ccRCC after progression on IO and VEGFR-TKI [25]. Preliminary efficacy endpoints included ORR of 50% and median PFS of 11.2 months. Toxicities were overall manageable with HTN (27%) and anemia (17%) being the most common grade 3–4 treatment-related adverse events (TRAEs). Additional trials with promising efficacy include a phase II study of batiraxcept (an AXL inhibitor) ± cabozantinib ± nivolumab (ORR 0% for batiraxcept monotherapy, but promising at 36% for batiraxcept with cabozantinib, and 55% for the triplet, respectively) and the phase I/II study of entinostat (histone deacetylase inhibitor) in combination with atezolizumab and bevacizumab (up to ORR of 60% in IO-naïve cohort) [26, 27]. Other notable trials in progress include the phase 1b/2 study of triplet therapy with ^177^Lu-girentuximab (antibody-radioisotope targeting carbonic anhydrase IX, which is expressed in > 90% of ccRCC tumor cells) combined with cabozantinib and nivolumab, and the phase I/II study of belzutifan with or without palbociclib (CDK 4/6 inhibitor, LITESPARK-024) [28, 29].

Though the therapeutic options for ccRCC have made significant advances, little improvement has been made in the management of non-clear cell renal cell carcinoma (nccRCC), in part due to its rarity and heterogeneity (different tumor types with distinct biological entities). Several recent studies suggest combination regimens with IO/VEGFR-TKI have reasonable efficacy in nccRCC (such as NCT03635892 evaluating cabozantinib plus nivolumab) [30]. An update from the phase II KEYNOTE-B61 (NCT04704219) of lenvatinib 20 mg PO daily and pembrolizumab 400 mg IV every 6 weeks was presented at ASCO 2023 (*n *= 158, including 59% papillary and 18% chromophobe) [30–33]. Overall, ORR was achieved in 49% of the patients, with a DCR of 82%, median PFS of 17.9 months, and 75% of the responders retaining their response ≥ 12 months. OS was not reached. Adverse events (AEs) were overall similar to those observed in the phase III CLEAR study with no new safety concern observed [32–34]. As such, KEYNOTE-B61 represents the largest trial to investigate IO/VEGFR-TKI combination in patients with previously untreated nccRCC and demonstrates promising antitumor activity of pembrolizumab plus lenvatinib.

Additionally, the interim analysis from the single-arm, phase II trial of the triplet combination, cabozantinib/ipilimumab/nivolumab in treatment naïve patients with nccRCC (CaNI trial, NCT04413123) were presented [35]. 39 patients received treatment, and at a median follow-up of 10.4 months, ORR was 18% with DCR of 76%. Twelve-month PFS was 51% and 12-month OS was 79%. Similar to COSMIC-313, where the same triplet was given in ccRCC, there was significant toxicity (grade 3/4 74% with grade 3/4 liver toxicity of 36%) and therapy discontinuation rate (21%), potentially contributing to the suboptimal efficacy. Thus, there remains a need for alternative dosing of current triplet therapy, or novel, more tolerable, triplet combinations [35, 36].

## Conclusion

Many novel therapies across GU malignancies have transformed outcomes for patients with prostate, bladder, and kidney cancers. Targeted treatments and antibody drug conjugates are heralding an era of precision oncology. Optimizing treatment selection and sequencing of therapies remains a challenge as resistance mechanisms are uncovered.

## Data Availability

Not applicable.
